# The Men of Fifty

**Published:** 1918-07-06

**Authors:** 


### THE MEN OF FIFTY.

The logic of events is apt to assert itself, and this to the surprise sometimes of persons who had not hesitated at the premises. In other words, it is readily possible for principles to be accepted, under the sway perhaps of patriotism or some other emotion, without at the same time a full appreciation of the inevitable consequences which must follow the decision. In due time the bill is presented for payment, and the absence of foresight cannot modify the demand. What has happened is the natural and unavoidable consequence of the preliminary steps. It may not have been foreseen, but the issue was implicit in the early approval. A history of this order may, we think, be traced in some of the events which have followed the latest Military Service Act. Under the pressure of a great emergency announced by the responsible Government of the day, Parliament, and we think we may add the country generally, accepted the proposition that the liability for medical service should be imposed on men up to the age of fifty-one years. We have no quarrel with this decision, and we feel quite sure that public opinion continues to support it, seeing that the alternative is failure to sustain our armies in the field, faithlessness to our Allies, and sooner or later disaster to the national cause. Yet from the domestic point of view the decision was a most serious one. It meant necessarily that men who have attained a full measure of middle age, and whose personal, domestic, and business habits have become a fixed quantity, are liable to be carried away into new positions and set to new and unaccustomed duties. Their lives and their health are to lose the shelter and protection they have hitherto enjoyed, and are to undergo the risks which are inevitable when organisations that have lost the flexibility of youth are called upon to adapt themselves to a new environment. Some cynical person has remarked that when men are no longer young they should not change their habits even though these are bad habits. On the score of morals the proposition may be challenged, but there is a spice of physiological wisdom in it. The truth it conveys is that by the time men have reached middle life their capacity for adapting themselves to new surroundings has become a limited quantity.

We repeat, therefore, that the decision to take such men for Army service was a most serious one. Nothing but actual necessity could justify it. This was the fashion in which it was presented to the country by the Prime Minister, and he could scarcely have used more solemn words. The consequences of the decision are now becoming manifest. Homes and businesses are being broken up, and financial and other hardships are falling on individuals who have hitherto led an easy and undisturbed existence. In certain quarters this position is causing an outcry of indignation. For the small minority who opposed the extension of the military age such an outcry may be reasonable enough. But it is quite inappropriate to those who supported the extension. By their present complaints they merely announce that when they endorsed the Government's policy they had not sufficient wisdom to realise what this policy would mean in actual practice. The decision contained within itself the consequences of which they now make complaint. No one pretends to like these consequences. No one pretends to like the policy. We would all much prefer to dwell in peace under our own vine and fig-tree. But if the policy is once accepted as inevitable the consequences are inherent in it, and both reason and common sense say that they, too, must be accepted. Who wills the end wills also the means. In a word, it is simply impossible to call a large proportion of the middle-aged men of the country into the Army and at the same time to avoid a great and painful disturbance of the economic and domestic life of the country and much suffering and hardship to individuals. To wish it were otherwise is an idle thing. It is to ignore the inevitable logic of events.

To approve a policy, however, and to foresee its broad and general consequences does not involve approval of all the detailed application of such policy in practice. And it is in this field that helpful criticism has its opportunity. The administration of the new Military Service Act is the business of the Ministry of National Service, and it has not been altogether a fortunate and happy one. We agree that some of the complaints advanced merely show that the persons who utter them fail to realise the full meaning of the vote which they gave in an earlier day. But there are others which may not be dismissed so lightly. It is undeniable that there is wide discontent with the interpretation that has been placed on the grading carried out by the medical boards, and when the esteemed President of one of the most important of the Tribunals under the Act announces openly that he will read Grade I. as meaning Grade II., no one can doubt that the discontent has a substantial foundation. The position has not been mended by some rather bitter words which escaped from the Minister of National Service, but as he has now made due apology for these it would be ungenerous to insist on them. What the country wants to know is that men who are not physically fit for active service shall not be marked by a sign which indicates a full degree of capacity, and which, so far as we can gather, the Army authorities interpret in this sense. There is a strong desire also to make sure that the military arrangements shall not be allowed to ignore the classification which has been fixed by the lay tribunals, and there is a considerable suspicion of the War Office in this respect. We believe that the opportunities for medical review of every preliminary decision are adequate, but they ought to be made more widely known, and were there not evidence to the contrary we should have refused to believe that any medical board could have been concerned in an invitation to examined men to register approval of the methods of examination. There are other points which await criticism, but in the meantime we postpone these until the new administrative arrangements have come into operation.

